# Energy Expenditure of a Single Sit-to-Stand Movement with Slow Versus Normal Speed Using the Different Frequency Accumulation Method

**DOI:** 10.3390/medicina55030077

**Published:** 2019-03-25

**Authors:** Takashi Nakagata, Yosuke Yamada, Yoichi Hatamoto, Hisashi Naito

**Affiliations:** 1 Faculty of Health and Sports Science, Juntendo University, Hiraka-gakuendai 1-1, Inzai, Chiba 270-1695, Japan; hnaitou@juntendo.ac.jp; 2 National Institute of Health and Nutrition, National Institutes of Biomedical Innovation, Health and Nutrition, 1-23-1 Toyama, Shinjuku-ku, Tokyo 162-8636, Japan; yamaday@nibiohn.go.jp (Y.Y.); monkeymatuyama@yahoo.co.jp (Y.H.); 3 Sportology Center, Juntendo University Graduate School of Medicine. 2-1-1, Hongo, Bunkyo-ku, Tokyo 113-8421, Japan; 4 The Fukuoka University Institute for Physical Activity, 8-19-1 Nanakuma, Jonan-ku, Fukuoka 814-0180, Japan

**Keywords:** sedentary break, non-exercise activity thermogenesis (NEAT), stand up, health promotion, population strategy, weight control, energy cost, home-based exercise

## Abstract

*Background and objectives:* The purpose of this study was to compare the energy expenditures (EE) of a single sit-to-stand (STS) movements with slow and normal speeds using a multi-stage exercise test. *Materials and Methods:* Twelve young males, aged 21–27 years (age, 23.0 ± 1.7 years; height, 171.2 ± 6.1 cm; weight, 64.3 ± 5.6 kg), performed repeated 3-s stand-up and 3-s sit-down (slow) or 1-s stand-up and 1-s sit-down (normal) movement on two different days with random order. All the participants completed multi-stage tests at different STS frequencies per minute. The slope and intercept of the linear regression relationship between the EE (kcal/min) and the STS frequency were obtained, and the slope of the regression was quantified as the EE of an STS. *Results:* The metabolic equivalents (METs) of the STS-slow was 4.5 METs for the frequency of 10 times/min (in total 1 min), and the net EE was 5.00 ± 1.2 kcal/min. The net EE of the STS-slow was 0.37 ± 0.12 kcal, which was significantly greater than that during the STS-normal (0.26 ± 0.06 kcal). The difference between the EEs of the STS-slow and STS-normal was significantly greater in taller and heavier subjects. *Conclusions:* We concluded that the intensity of STS-slow movement is moderate, and the EE during an STS-slow (0.37 ± 0.12 kcal) is higher than that during an STS-normal (0.26 ± 0.06 kcal). Our study results will help exercise and/or health professionals prescribe physical activity programs using STS movement for healthy young population groups.

## 1. Introduction

Prolonged unbroken sitting time (e.g., watching TV at home and work at the office) is related to the risk of various disease outcomes in healthy people, including obesity, type 2 diabetes, cardiovascular disease, and all-cause mortality [1,2,3,4,5]. Performing physical activity including non-exercise activity thermogenesis (NEAT) [6] and increasing daily total energy expenditure (EE) have contributed significantly to health outcomes [7]. Replacing prolonged sitting time with standing time has improved in cardiometabolic risk factors [8,9], however, a previous study indicated that the EE of standing posture per se is not much different from the EE of sitting position [10].

In contrast, the EE of a sit-to-stand (STS) movement and alternating positions, which is performed many times in daily life, is higher than in sitting and standing [11,12,13]. For example, Judice et al. demonstrated that the EE of a single sit-to-stand transition was 1.49 ± 0.2 kcal/min and the energy cost was 0.32 kcal (35% above sitting). Furthermore, The EE and exercise intensity of STS can be easily adjusted by changing the seat height [14,15], and the *V*O_2_ during the STS increased as the seat height decreased [16]. Therefore, increasing EE by repeating the STS and changing the height of the seat may be a simple physical activity and/or exercise that has a beneficial effect on health outcomes.

In order to measure EE during physical activity performed within a few seconds, Hatamoto et al. [17,18] developed a novel method, named the “Different Frequency Accumulation Method (DFAM)”. The DFAM is a graded multi-stage exercise test consisting of four-minute in each stage, during each of which participants repeat the same exercise at different frequencies; EE for a single movement (energy cost) in the exercise is then estimated using the linear relationship between gross EE and the frequency with which the movement is performed. They reported that the energy cost of the STS in 1 s was 0.22 ± 0.09 kcal using DFAM [19]. In addition, they examined the relationship with the EE and anthropometric characteristics (body height, weight), and demonstrated that the tallest participant’s EE for an STS movement was more than triple compared with the shortest participant. However, they investigated the EE of STS at only one speed (1s up, 1s down). The STS movement requires thigh and hip muscle extension and is performed as a chair stand exercise for low-fitness and/or older populations with limited mobility [20], and a previous study has reported that performing a body weight resistance exercise including chair stand exercises with slow movement (3 s up, 3 s down) improves physical functions in comparison to that being performed at normal speed (1 s up, 1 s down) in older subjects [21]. We hypothesized that the EE of STS depends on the movement speed of STS, but no study has been conducted to compare the EE of a single STS with slow and normal body movements of STS.

The purpose of this study was (1) to compare the energy expenditure of STS with normal and slow movements using the Different Frequency Accumulation Method (DFAM), and (2) to examine the relationship between the EE of a single STS with height and weight.

## 2. Materials and Methods

### 2.1. Participants

This study included 12 adult males aged 21–27 years (age, 23.0 ± 1.7 years; height, 171.2 ± 6.1 cm; weight, 64.3 ± 5.6 kg). Prior to the study, all the participants provided written consent to participate after receiving information about the procedures and purpose of the study. The study protocol was approved by the Research Ethics Review Board of the Juntendo University Graduate School of Health and Sports Science (28–25).

### 2.2. Experimental Design

The study was of a random crossover design with two conditions, including slow and normal movements on two separate days. All the measurements were carried out in a laboratory where the temperature and humidity of the internal atmosphere were adjusted to 20 °C and 50%, respectively. All the participants completed both the experiments on two separate days within one week between October 2016 and April 2018.

The participants in this study refrained from any strenuous physical activity, including general exercise, from the day before starting the experiment, and started fasting (no water restriction) 4 h before starting the experiment. The height and body weight were measured before exercise. For each participant, the REE was measured in both the slow and normal movement sessions using an indirect calorimeter (AE-300s, Minato Medical Science Co., Ltd., Osaka, Japan) while sitting on a chair and maintaining a resting position for 20 min with a face mask attached. After measurement of their REE, each participant carried out an exercise session. In session 1, they performed either the slow or normal movements sessions. In session 2, they performed either the slow or normal movements. In order to eliminate influences of execution order, the allocation of the slow or normal movements to session 1 and session 2 was randomized between participants.

### 2.3. Sit-to-Stand Movement

**Figure 1:**
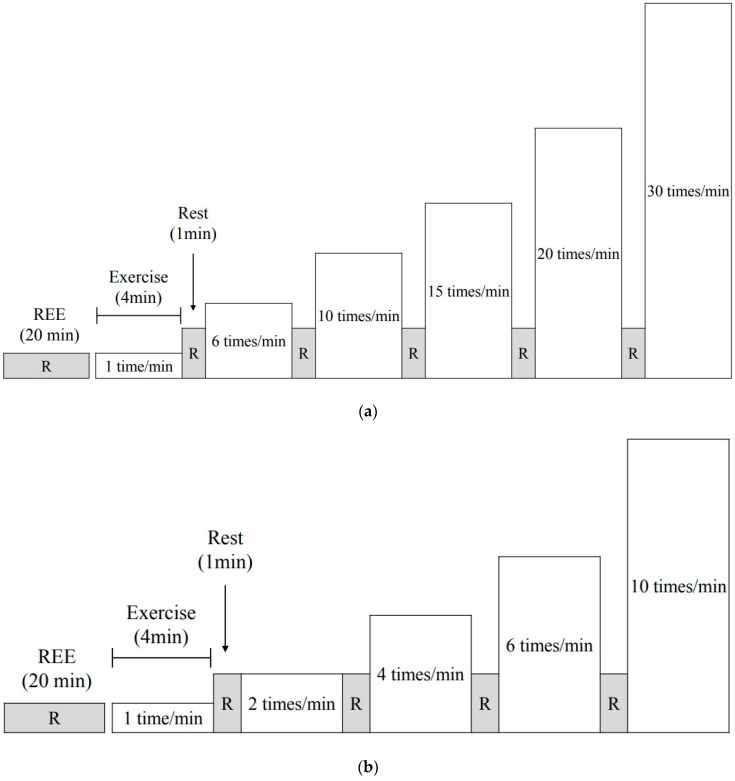
Experimental protocol. (**a**) The sit-to-stand movement (STS) with normal speed protocol consisted of six incremental stages. The STS frequencies of each stage were 1, 6, 10, 15, 20, and 30 times/min. (**b**) The STS with slow speed protocol consisted of five incremental stages. The STS frequencies of each stage were 1, 2, 4, 6, and 10 times/min. REE: resting energy expenditure.

The experimental protocol for all participants is shown in Figure 1a,b. The participants completed the multi-stage experiment at different STS frequencies. The participants adjusted the rhythm with the sound of a metronome. The chair used in our study was 40 cm tall, 44.0 cm wide, 41.0 cm long, and armless and backless. All participants performed sit-to-stand movements with both speeds to familiarize themselves with the experimental protocol before the main experiments.

### 2.4. Indirect Calorimetry Measurement and Energy Expenditure

Respiratory gas measurement was carried out using indirect calorimetry in our laboratory as previously described [22]. The last 2 min in each stage was used to evaluate the oxygen uptake (*V*O_2_) and carbon dioxide production (*V*CO_2_), and the EE was calculated using the Weir equation [23] for *V*O_2_ (L/min), and *V*CO_2_ (L/min). During the resting period of 20 min, the average value during the last 10 min was defined as the individual’s REE. The *V*O_2_ of 3.5 mL/kg/min was designated as 1 MET.

### 2.5. Different Frequency Accumulation Method (DFAM)

Calculating the EE was modeled from a previous study [19]. The DFAM is the idea that gross EE increases linearly as movement frequency increases if the EE, which is determined for performing one movement, accumulates by conducting movements repeatedly. The linear relationship between the gross EEs and different frequencies of the movement indicated that the slope of the regression line was expressed as an EE of a movement (net EE). In addition, the intercept (a zero-load EE) meant an individual REE was also included in the linear regression analysis.

### 2.6. Heart Rate, Ratings of Perceived Exertion, and Blood Lactate Concentration

Heart rate (HR) was recorded during the whole experiment using an electrocardiogram device (Fukuda Electronics Co., Ltd. Tokyo, Japan). Three beats were recorded 15 s before the end of each stage; the average value was taken as the HR of each stage. The rate of perceived exertion (RPE) was recorded using a 6–20 steps Borg scale [24] after each stage. Blood samples (20 μL) were collected from the earlobe using a capillary tube [25] using a Biosen S-Line device (EKF Diagnostik, Barleban, Germany) before the exercise and immediately after each stage in both experiments.

### 2.7. Statistical Analyses

Microsoft Office Excel 2017 and PASW Statistics version 20.0 (SPSS, IBM Inc. BM Corp., Armonk, NY, USA) were used for data processing and statistical analyses, respectively. All the variable results are presented as mean ± standard deviation. The linear regression analyses were performed to calculate the slopes and intercepts for the gross EE against the STS frequencies. A paired t-test was conducted to compare the slope, the intercept, and the REE. The statistical significance level was set at 0.05.

## 3. Results

All participants successfully completed both experimental sessions, and they could maintain the rhythms of the metronome during experiments.

The individual’s resting *V*O_2_ and HR for the sitting position were not significantly different in both experiments (*V*O_2_; normal: 4.2 ± 0.3 mL/kg/min vs. slow: 4.1 ± 0.3 mL/kg/min, *p* = 0.562, HR; normal: 66 ± 11 bpm vs. slow: 65 ± 13 bpm, *p* = 0.666).

**Figure 2:**
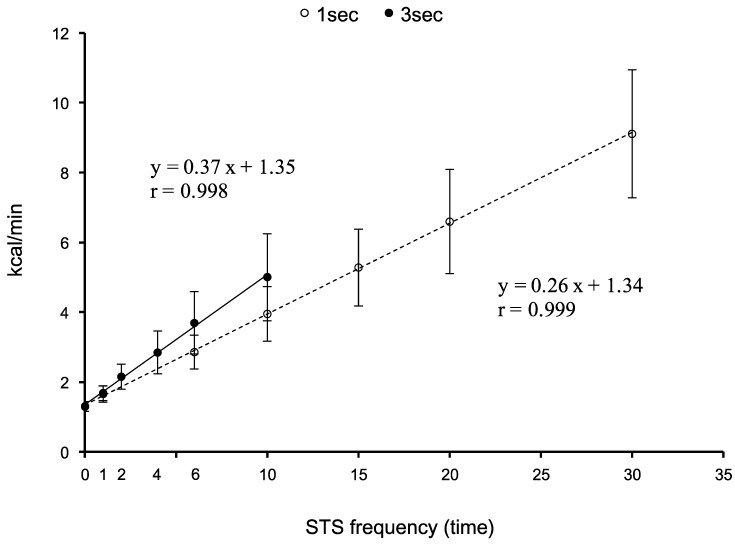
The linear regression relationship between gross energy expenditures (EE) (kcal/min) and the STS frequency (times/min). The symbol “**white circle**” is STS-normal, and the symbol “**black circle**” is STS-slow. The STS frequency was 1, 6, 10, 15, 20, and 30 times/min (normal), and 1, 2, 4, 6 and 10 times/min (slow). The slope of the STS-slow was significantly higher than that of the STS-normal (slope; 0.37 ± 0.12 vs. 0.26 ± 0.06 kcal, *p* < 0.001).

Figure 2 shows the relationship between an STS frequency and the gross EE for both experiments. The gross EE increased linearly against the STS frequencies for both the slow and normal movements; the slope of the regression of the slow movement was higher than that of the normal movement (slope; 0.37 ± 0.12 vs. 0.26 ± 0.06 kcal, *p* < 0.001). The value of an intercept in the regression (equivalent to gross EE in a sitting position) did not significantly differ between slow and normal movements.

Table 1 shows the relationship between an STS frequency and gross EE, METs, HR, RPE, and La during both the STS movements at each stage. All the variables were higher during the slow movement at a common STS frequency (1, 6 and 10 times/min). The METs of the STS-slow at the frequency of 10 times/min (total in 1 min) was 4.5 METs, and the net EE was 5.0 kcal/min; the METs and EE were significantly higher at 30 times/min (in total 1 min) in STS-normal as compared to STS-slow.

**Figure 3:**
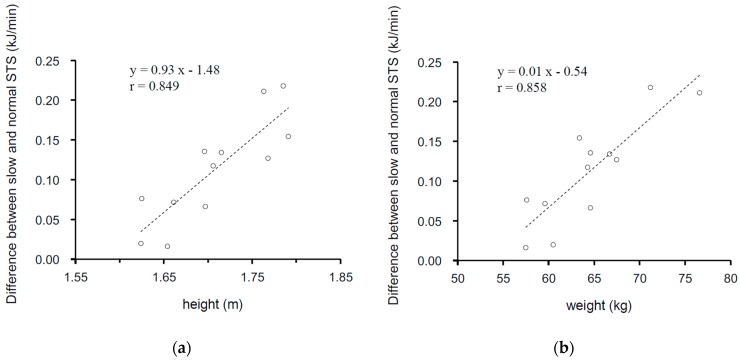
The relationship between the difference of EE (kcal/min) between slow and normal STS movement. (**a**) Height (m) and (**b**) body weight (kg) are shown. Each plot was the difference in the EE of an STS (STS-slow—STS-normal) of all participants.

Figure 3 shows the relationship between anthropometric characteristics and the EE of an STS. The EE of an STS increases as a participant’s height (r = 0.849, *p* < 0.001) and body mass increase (r = 0.858, *p* < 0.001).

## 4. Discussion

In this study, we examined the EEs during the STS movements with slow and normal speeds using a multi-stage exercise test named the Different Frequency Accumulation Method (DFAM). We found that the net EE of an STS-slow is approximately 40% larger than that of the normal movement (slope; 0.37 ± 0.12 vs 0.26 ± 0.06 kcal). The difference between the EEs of the STS-slow and STS-normal were significantly greater in taller and heavier subjects.

The gross EE (kcal/min) increased linearly against the STS frequencies for both the slow and normal speeds (slow: y = 0.37 + 1.35, r = 0.998; normal: y = 0.26 + 1.34, r = 0.999, Figure 2). Furthermore, the gross EE of the STS frequencies calculated using DFAM was almost the same value as the actual EE at 1/times (Table 1). With regard to exercise intensity (METs), the average METs for 10 times/min of STS-slow (in total 1 min) and STS-normal (in total 20 s) were 4.5 ± 0.7 Mets and 3.6 ± 0.4 Mets, respectively; those METs values were equivalent to moderate intensity exercises such as walking for leisure (3.5 METs) and brisk walking at 5.6 km/h (4.3 METs) [26]. Furthermore, the METs for 10 times/min of STS-slow were almost the same value as the 15 times/min of STS-normal (slow; 4.5 METs, normal; 4.8 METs). The HR and RPE of the STS movement of both speeds except for STS-normal for 30 times/min were categorized to light to moderate intensity, and blood lactate concentration was less than 2 mmol/L. Therefore, with regard to METs, HR, RPE, and blood lactate concentration, which is considered when performing daily physical activity and prescribing an exercise program, repetitive STS with slow and normal (~20 times/min) movements may be termed as a moderately intense physical activity [27]. Furthermore, when comparing the slope of both experiments, the slope of the STS-slow was significantly greater than that of the STS-normal; the slope of the STS-slow was approximately 40% larger than that of the STS-normal (slow: 0.37 ± 0.12 kcal; normal: 0.26 ± 0.06 kcal). However, this result is not surprising, because at the time of muscle contraction, the STS-slow tripled (from 1–3 s).

We also examined the relationship between the EE of an STS with respect to the height and weight of the participants. The EE of an STS increased as the body height and weight increased during both experiments. Furthermore, as shown in Figure 3, the difference between the EEs of the slow and normal speeds was significantly greater in taller subjects and heavier subjects (Height; r = 0.849, Weight; r = 0.858). When using the same chair with a height of 40 cm, regardless of the subjects’ heights, taller people needed to make larger movements when performing the transitions from sitting to standing. A previous study indicated that a lower seat brings down the center of gravity and increases the degree of trunk flexion and angular displacement of the trunk, hip, knee, and ankle [28], and the EE during an STS increases while standing up from a lower seat [16]. Therefore, when an individual is taller or heavier, the load on the body increases, and it becomes necessary to have greater energy.

Recent physical activity guidelines recommend performing brief bouts (<5 min) of physical activity, including non-exercise physical activity thermogenesis (NEAT), such as standing and walking around [29,30]. However, it may be quite difficult for everyone to engage in walking/jogging exercise in real-world situations. An STS is a physical activity that the majority of individuals can perform easily anywhere without any special tools or spaces, and without breaking a sweat.

We investigated a single EE (energy cost) of STS movements performed at different speeds using the DFAM; however, this study has several limitations. First, all the study participants were healthy young males. STS movements with slow and normal speeds were light- to moderate-intensity, at least for healthy young men, but the intensity of STS movement will become relatively higher for other populations. In particular, older adults expend more energy during simple walking at 5 speeds between 0.7 and 1.8 m/s [31], and previous studies reported higher muscle coactivation during the postural control in older as compared to younger adults using electromyography (EMG) analysis [32,33]. Therefore, it is important that additional research is carried out in other populations (women and/or older adults) to investigate the influence of sex, age, and body compositions. Second, we investigated only two speeds of STS movement (slow: 3 s up, 3 s down; normal: 1 s up, 1 s down). In our daily lives, we perform STS movements at different speeds, and further studies are needed to apply this method of measuring the EE of STS movements at different speeds (e.g., more slowly or more quickly). Third, our study did not measure muscle activity using EMG analyses and muscle oxygenation level using near-infrared spectroscopic (NIRS) analyses, and therefore, it is unclear what the differences of the metabolic responses in muscles between slow and normal speeds are. Although we did not measure EMG nor NIRS in this study, previous studies reported that low-intensity knee extension exercises with slow movement make muscle tissue environment different from knee extension exercises with normal movements [34]. Further research is needed to validate these findings combining respiratory gas measurement, EMG, and NIRS analyses.

## 5. Conclusions

This study compared the EE of the STS movements performed at different speeds; the EE of the STS-slow was 40% greater than that of the STS-normal, and EE of an STS increased as the height and body weight increased at both speeds. Furthermore, the exercise intensity of the STS-slow was 4.5 METs at the frequency of 10 times/min (in total 1 min) on an average. The results of our study are valuable for exercise/health professionals to help prescribe exercise programs using STS for healthy young population groups.

## Figures and Tables

**Table 1:** The relationship between the frequency of STS and physiological responses both for normal- and slow-speed movements.

Normal (1 s)	1 time/min	6 times/min	10 times/min	15 times/min	20 times/min	30 times/min
Exercise timing	0 (every 1 min)	0,10,20, 30,40,50 (every 10 s)	0,6,12… 48,54 (every 6 s)	0,4,8… 52,56 (every 4 s)	0,3,6… 54,57 (every 3 s)	0,2,4… 56,58 (every 2 s)
EE (kcal/min)	1.7 ± 0.2	2.9 ± 0.5	4.0 ± 0.8	5.3 ± 1.1	6.6 ± 1.5	9.1 ± 1.8
METs	1.5 ± 0.2	2.7 ± 0.3	3.6 ± 0.4	4.8 ± 0.6	5.9 ± 0.8	8.1 ± 0.9
HR (bpm)	79 ± 10	86 ± 10	95 ± 13	103 ± 13	118 ± 15	137 ± 25
RPE	7 ± 1	9 ± 2	10 ± 2	11 ± 2	12 ± 2	14 ± 3
La (mM)	0.68 ± 0.20	0.71 ± 0.22	0.74 ± 0.24	0.84 ± 0.46	1.23 ± 0.86	2.48 ± 1.47
**Slow** **(3 s)**	**1** **time/min**	**2** **times/min**	**4** **times/min**	**6** **times/min**	**10** **times/min**	
Exercise timing	0 (every 1 min)	0,30 (every 30 s)	0,15, 30,45 (every 15 s)	0,10,20, 30,40,50 (every 10 s)	0,6,12… 48,54 (every 6 s)	
EE (kcal/min)	1.7 ± 0.2	2.2 ± 0.4	2.8 ± 0.6	3.7 ± 0.9	5.0 ± 1.2	
METs	1.5 ± 0.2	2.0 ± 0.2	2.6 ± 0.3	3.3 ± 0.5	4.5 ± 0.7	
HR (bpm)	80 ± 10	83 ± 10	86 ± 11	92 ± 13	101 ± 13	
RPE	7 ± 2	8 ± 2	9 ± 2	10 ± 3	11 ± 3	
La (mmol/L)	0.90 ± 0.26	0.87 ± 0.18	0.85 ± 0.21	0.90 ± 0.23	1.47 ± 0.97	

Note: METs, metabolic equivalents; HR, heart rate; RPE, ratings of perceived exertion; La, blood lactate.
